# Dietary patterns and oral health practices among primary school children aged 6–15 years in urban informal settlements of Eldoret City, Kenya

**DOI:** 10.1186/s12903-026-08778-6

**Published:** 2026-06-25

**Authors:** Florence Wandia, Joel Wanzala

**Affiliations:** School of Health Sciences, University of Kabianga, Kericho, Kericho County, Kenya

**Keywords:** Dietary patterns, Oral health practices, Dental caries, School children, Informal settlement, Kenya

## Abstract

**Background:**

Dental caries remains one of the most prevalent chronic conditions among school-aged children, disproportionately affecting those in informal settlements where dietary risks and preventive oral health services are limited.

**Aim:**

This study assessed dietary patterns, oral hygiene practices, and their association with dental caries among primary school children aged 6–15 years in informal settlements of Eldoret City in Uasin Gishu County, Kenya.

**Methods:**

The study adopted a school-based cross-sectional design and involved 407 primary school children selected using proportionate stratified random sampling. Data was collected using a validated and pretested structured questionnaire, while clinical oral assessment was based on WHO field guidelines for the examination of dental caries. Data was analyzed using SPSS v.26. Chi-square tests assessed associations, and bivariate and multivariate logistic regression models were used to identify independent dietary patterns and oral hygiene practices associated with dental caries.

**Results:**

Out of 407 children, 55.3% were females, and their average mean age was 12.15 ± 2.98 years. The prevalence of dental caries was 26.0% (95% CI: 21.8–30.3). Daily consumption of sugary foods and beverages and suboptimal oral hygiene practices were relatively high. Multivariate logistic regression models revealed daily intake of biscuits, cakes, sweet pies, and buns (AOR = 2.31; 95% CI: 1.36–3.74; *p* = 0.004), daily consumption of sweets and candy (AOR = 2.42; 95% CI: 1.35–3.91; *p* = 0.006), brushing less than twice daily (AOR = 2.92; 95% CI: 1.18–3.87; *p* = 0.023), and not using fluoride toothpaste (AOR = 1.83; 95% CI: 1.12–2.61; *p* = 0.043) were independently associated with higher odds of dental caries among primary school children.

**Conclusion:**

Integrated school- and community-based prevention strategies targeting dietary patterns and oral hygiene promotion are urgently needed to reduce preventable oral health inequities among vulnerable schoolchildren.

## Introduction

Nutrition and oral health are significant public health issues and components of overall health and well-being globally, particularly among children [1]. Oral diseases are one of the most prevalent non-communicable conditions that affect school-aged children globally, with dental caries identified as the single most prevalent chronic disease in childhood [2]. Approximately two billion people live with oral diseases [dental caries] in permanent teeth worldwide, while untreated caries in deciduous teeth affect approximately 600 million children [1]. The burden of oral diseases persists despite being largely preventable through adequate and appropriate nutritional and oral hygiene practices [3]. Poor dietary patterns, particularly the frequent consumption of free sugars and refined carbohydrates, remain one of the major modifiable risk factors for dental caries [4]. Poor diet and oral hygiene practices contribute to oral diseases due to acid production by oral biofilms that demineralize tooth enamel [5, 6].

Schoolchildren aged 6–15 years are particularly vulnerable to dental caries and other oral health problems [7, 8]. This age represents a critical developmental stage for oral health, as it entails mixed dentition and development of permanent teeth [9]. This heightens vulnerability to cariogenic exposures and oral health practices that can impact a child’s oral health and quality of life (QoL) [10]. According to Hazara [6], poor dietary patterns and oral health in this age group have significant immediate and long-term consequences such as nutritional and eating difficulties, pain, infections, disrupted sleep, discomfort, diminished self-esteem, and school absenteeism. These contribute to poor educational performance and QoL. Aljafari et al. [8] emphasize that poor dietary patterns, such as regular intake of sugar-sweetened snacks and beverages, and inadequate oral health practices, such as irregular toothbrushing with fluoride toothpaste, in childhood often persist into adolescence and adulthood. As a result, these increase the risk of chronic systemic conditions such as diabetes and obesity.

The burden of oral diseases among children remains inadequately characterized and poorly controlled in sub-Saharan Africa, particularly in slum settings, which are characterized by inadequate and inaccessible health facilities such as oral and nutrition services [11]. Studies conducted in urban informal settlement settings have consistently reported a high burden of untreated oral diseases and progress to more severe stages among school-aged children due to suboptimal oral hygiene and unhealthy dietary practices [12, 13]. These are exacerbated by structural barriers such as poverty, overcrowding, limited caregiver oral health literacy, and weak integration of preventive oral health services within primary health and school health systems [11, 12]. Despite this evidence, there remains a critical gap in empirical and knowledge data focusing on dietary patterns and oral health practices among primary school-aged children in urban informal settlement settings in Kenya, particularly in Uasin Gishu County. Existing studies have mainly focused on preschool populations in urban areas [14], leaving out informal or slum settings and/or integrating dietary intake and a broader spectrum of oral hygiene practices. This gap presents a barrier to informed public health planning, as school environments and family feeding practices have profound implications for modifiable risk exposures during formative years, particularly in urban informal settlements. Therefore, this study aimed to assess dietary patterns and oral health practices among primary school children aged 6–15 years in urban informal settlements of Eldoret City, Kenya, using a cross-sectional design. The study is critical to inform effective health promotion interventions and preventive strategies such as the Kenya National Oral Health Strategic Plan 2022–2026.

## Methodology

### Study design and setting

The study employed a school-based cross-sectional analytical study design. The design enabled effective assessment of dietary patterns and oral hygiene practices among school-going children aged 6–15 years at a single point in time, while allowing the exploration of associations. Existing studies focused on dietary behaviours and/or oral health practices have adopted similar designs [4, 10]. The study was conducted from September to October 2025 in two selected primary schools within the Langas (Langas Primary School) and Ngomongo (Bomongo Primary School) informal settlements in Eldoret City, Uasin Gishu County. These informal settlements are characterized by high population density, low socioeconomic status, constrained or inaccessible preventive health services [11], and reliance on unhealthy, inexpensive, sugar-dense diets, which might contribute to low adherence to WHO-recommended oral hygiene practices and dietary patterns.

### Study population

The study population comprised primary school children aged 6–15 years enrolled in lower and upper primary classes within the selected schools. The study focused on this age group to capture children in the mixed and early permanent dentition dynamics. This age group is critical for the establishment of dietary habits and oral hygiene practices that have immediate and long-term oral health outcomes [15]. The study included children aged 6–15 years who were present during data collection and whose parents or guardians provided informed consent alongside child assent, while children with pre-existing dental disorders or conditions, undergoing orthodontic treatment, or absent were excluded from the study.

### Size and sampling

The sample size was determined using Cochran’s formula, n₀ = (Z² X p X (1-p))/e² [16]. Where n₀ = required sample size, Z = Z-value (1.96 for a 95% confidence level), p = estimated proportion of the population with oral health issues (assumed to be 0.5, as there is no prior estimate), and e = margin of error (set at 0.05), resulting in 384. To account for design effect and potential nonresponse, 10% was added; hence, a total sample of 422 children was targeted. A multistage sampling technique was used. In the first stage, Langas and Ngomongo informal settlements were purposively selected due to their urban informal settlement characteristics and study focus. In the second stage, a list of all eligible primary schools within each settlement was obtained from the Uasin Gishu County Education Office. Since each settlement had two primary schools, one school per settlement was selected using simple random sampling. Probability proportionate to size sampling (PPS) was used to sample size of each selected primary school based on estimated samples. In the final stage, eligible children were selected from class registers using proportionate stratified random sampling to ensure representation across age groups and grade levels.

### Data collection tools and procedure

Data were collected using a structured, interviewer-administered questionnaire adapted and developed and customized based on the literature that assessed diet and oral health and nutritional knowledge among school-going children [17, 18]. Validated and internationally recognized tools, particularly the WHO simplified oral hygiene index (OHI-S) questionnaire for children and previously validated school-based dietary assessment tools used in LMICs, were adapted [17, 18]. The questionnaire consisted of three major sections, namely, sociodemographic characteristics, oral health practices, and dietary pattern assessment. The sociodemographic section entailed age, gender, grade (class), residence, religion, parental education level, parental employment status, and household income. This section was given to eligible children to fill out with the help of a parent or legal guardian at home before returning it to the researcher the following day at school. Prior to handing over, the rest of the sections were administered to the children with the help of trained research assistants and pinned together. Oral health practices were assessed using the WHO-OHIS questionnaire, which consisted of 14 items focused on self-reported frequency and timing of toothbrushing, use of fluoride toothpaste, history of dental discomfort or complications, and dental service utilization [17]. Minor contextual adaptations were made to fit local practices while preserving the content validity of the tool. A child’s dietary patterns were assessed using the Children’s Dietary Questionnaire (CDQ) [19]. The CDQ assessed the usual weekly frequency of consumption of contextually selected foods relevant to oral health, such as desserts and confectionery (biscuits, sugary candies, sweets, cakes, sweet pies, and buns); sweet drinks (sugar-sweetened beverages, fruit juices such as orange juice and apple juice, carbonated soft drinks such as cola, and non-fresh fruit juices such as lemonade); sugar-sweetened milk (yogurt); vegetables; and fresh fruits. Data collection was conducted during designated school hours by trained research assistants using standardized procedures. The selected children, particularly the younger child, received clarifications from the interviewer to minimize potential comprehension and recall bias. The interviews were conducted in private settings within the school environment designated by school administration to promote confidentiality and privacy and minimize social desirability bias.

Clinical examinations were performed to determine the presence or absence of dental caries. The examination was carried out in school by two experienced dentists under natural light. A plane mirror and dental probe were used to assess dental caries. Based on WHO diagnostic criteria [17], dental caries was only recorded if there was a visible break in the enamel or marked shadow under the enamel of enrolled children. The enamel was classified as healthy if there was any doubt.

### Data quality assurance

The questionnaire was developed in English, translated into Kiswahili, and back-translated to ensure semantic equivalence. The pilot study was conducted two weeks prior to the main study to ensure readability, relevance, and context-specific tailored study tools, with revisions made prior to field deployment. A pilot study involved 50 school-going children aged 6–16 years at Munyaka Primary School, located within informal settlements of Eldoret City in Uasin Gishu County. This school did not form part of the main study population. This pilot study was conducted to assess the internal reliability of the questionnaire. The internal reliability test yield a Cronbach’s alpha coefficient of 0.83, indicating the items have acceptable internal consistency. The research assistants underwent five days of intensive training on study objectives, ethical conduct, participant recruitment and engagement, child-appropriate interviewing techniques, and standardized administration of the questionnaires to reduce interviewer variability and bias. The content was reviewed by two oral health experts, three nutrition experts, and one public health expert to ensure content relevance. Completeness of filled questionnaires was assessed after completion of each interview. Double data entry and validation checks were performed to reduce transcription errors. Standardized coding strategies were applied consistently.

### Data management and statistical analysis

Completed questionnaires were reviewed in the field for completeness and consistency before data entry. Missing data or incomplete questionnaires were excluded from analyses. Data were coded and entered into the Statistical Package for the Social Sciences (SPSS) version 26. Double-entry verification was performed to minimize data entry errors. Data cleaning procedures included range checks, assessment of missing values, and logical consistency checks prior to analysis. Descriptive statistics were used to summarize socio-demographic characteristics, dietary patterns, and oral health practices, with categorical variables presented as frequencies and percentages. Chi-square analysis was conducted to assess associations between dietary patterns and oral health practices and dental caries. To avoid premature exclusion of variables, all variables with a p-value < 0.10 in the chi-square analysis were entered into bivariate and multivariable logistic regression models. Regression models were employed to identify factors independently associated with poor oral health practices while adjusting for potential confounders such as parental education, income, and living arrangement. with results reported as adjusted odds ratios and corresponding 95% confidence intervals. Statistical significance was set at *p* < 0.05 for all analyses.

## Results

Out of 422 recruited school children, 407 (96.5%) fully completed the questionnaires. More than half of participants were females (55.3%). The average age of participants and number of their siblings were 12.15 ± 2.98 years and 3.23 ± 1.54, respectively. Similarly, the majority were in grade 5 (35.1%), lived with both parents (70.6%), and had parents who were informally employed (Table 1).

**Table 1:** Socio-demographic characteristics of school children enrolled in the study

Socio-demographic	*N* (%)
Gender	
Male	182 (44.7)
Female	225 (55.3)
Age	
Mean ± S.D.	12.15 ± 2.98
Number of Siblings	
Mean ± S.D.	3.23 ± 1.54
School Class	
Grade 4	93 (22.9)
Grade 5	143 (35.1)
Grade 6	87 (21.4)
Grade 7	84 (20.6)
Living arrangement	
Both Parent	288 (70.8)
Single Parent	97 (23.8)
Guardian/relatives	22 (5.4)
Father’s Occupation	
Formally Employed	103 (25.3)
Informally Employed	164 (40.3)
Unemployed	136 (33.4)
No Father/male in the household	4 (1.0)
Mother’s Occupation	
Formally Employed	63 (15.5)
Informally Employed	189 (46.4)
Unemployed	155 (38.1)
Father’s Education	
High	166 (40.8)
Moderate	98 (24.1)
Low	139 (34.2)
No male in the household	4 (1.0)
Mother’s Education	
High	138 (33.9)
Moderate	123 (30.2)
Low	146 (35.9)

Note: High = university or college, Moderate = secondary and/or high school, Low = Primary and/or no formal education; N = frequency, S.D. = standard deviation

Table 2 shows self-reported dietary patterns and dental caries among primary school children. Daily consumption of sugar-containing foods and beverages such as biscuits, cakes, cream cakes, sweet pies, buns, soft drinks (16.2%), jam/honey (16.7%), chewing gum containing sugar (17.9%), sweets/candy (18.7%), milk with sugar (24.3%), and tea with sugar (49.6%). The prevalence of dental caries was higher among children who were daily soft drink consumers (13.8%), daily tea consumers (14.0%), and daily milk with sugar consumers (11.3%). Chi-square analysis revealed significant associations between biscuits/cakes, soft drinks, sweets/candy, milk with sugar, and tea with sugar (*p* < 0.05).

**Table 2:** Sugary food and beverage consumption and dental caries among primary school children

Sugary food consumption	Total	Caries	Caries-Free	*p*-value
	*N* (%)	*N* (%)	*N* (%)	
Biscuits, cakes, cream cakes, sweet pies, buns, etc.				0.016*
≤ 1 times/week	105 (25.8)	10 (2.5)	95 (23.3)	
2–6 times/week	218 (53.6)	46 (11.3)	172 (42.3)	
Daily	84 (20.6)	50 (12.3)	34 (8.4)	
Lemonade, Coca Cola or other soft drinks				0.035*
≤ 1 times/week	114 (28.0)	9 (2.2)	105 (25.8)	
2–6 times/week	227 (55.8)	41 (10.1)	186 (45.7)	
Daily	66 (16.2)	56 (13.8)	10 (2.5)	
Jam/honey				0.264
≤ 1 times/week	142 (34.9)	36 (8.8)	106 (26.0)	
2–6 times/week	197 (48.4)	48 (11.5)	150 (36.9)	
Daily	68 (16.7)	23 (5.7)	45 (11.1)	
Chewing gum containing sugar				0.113
≤ 1 times/week	109 (26.8)	24 (5.9)	85 (20.9)	
2–6 times/week	225 (55.3)	56 (13.8)	169 (41.5)	
Daily	73 (17.9)	26 (6.4)	47 (11.5)	
Sweets/candy				0.034*
≤ 1 times/week	114 (28.0)	30 (7.4)	84 (20.6)	
2–6 times/week	217 (53.3)	34 (8.4)	183 (45.0)	
Daily	76 (18.7)	42 (10.3)	34 (8.4)	
Milk with sugar				< 0.001*
≤ 1 times/week	84 (20.6)	28 (6.9)	56 (13.8)	
2–6 times/week	224 (55.0)	32 (7.9)	192 (47.2)	
Daily	99 (24.3)	46 (11.3)	53 (13.0)	
Tea with sugar				0.014*
≤ 1 times/week	87 (21.4)	15 (3.7)	72 (17.7)	
2–6 times/week	118 (29.0)	34 (8.4)	84 (20.6)	
Daily	202 (49.6)	57 (14.0)	145 (35.6)	
Coffee with sugar				0.291
≤ 1 times/week	104 (25.6)	26 (6.4)	78 (19.2)	
2–6 times/week	210 (51.5)	50 (12.3)	160 (39.3)	
Daily	93 (22.9)	30 (7.4)	63 (15.5)	

Note: Asterisk (*) Significant to the chi-square test (*p* < 0.05); N = Frequency

Table 3 shows that suboptimal oral hygiene practices were common among primary school children. Almost two-thirds (64.4%) brushed less than twice daily, 56.3% did not use toothpaste, 69.0% did not use fluoride toothpaste, 66.1% brushed only either in the morning or evening (once daily), and more than a half (58.2%) never visited the dentist or oral health officer in the previous 12 months. The prevalence of dental caries was higher among children who brushed less than twice daily (17.4%), did not use fluoride toothpaste (18.9%), and brushed once daily (17.4%). Chi-square analysis revealed significant associations between teeth-cleaning frequency, fluoride toothpaste utilization, and timing of brushing with dental caries among primary school children (*p* < 0.05).

**Table 3:** Oral hygiene practices and dental caries among primary school children

Oral Health Practices	Total	Caries	Caries-Free	*p*-value
	*N* (%)	*N* (%)	*N* (%)	
Teeth cleaning frequency				0.029*
≥ 2 times/day	145 (35.6)	35 (8.6)	110 (27.0)	
< 2 times/day	262 (64.4)	71 (17.4)	191 (46.9)	
Owned Toothbrush				0.17
Yes	391 (96.1)	92 (22.6)	299 (73.5)	
No	16 (3.9)	14 (3.4)	2 (0.5)	
Toothpaste Use				0.26
Yes	178 (43.7)	37 (9.1)	141 (34.6)	
No	229 (56.3)	69 (16.9)	160 (39.3)	
Fluoride Toothpaste Use				0.021*
Yes	126 (31.0)	29 (7.1)	97 (23.8)	
No	281 (69.0)	77 (18.9)	204 (50.1)	
Timing of Brushing				0.046*
Morning & Evening	138 (33.9)	35 (8.6)	103 (25.3)	
Morning or Evening only	269 (66.1)	71 (17.4)	198 (48.6)	
Dental visits past 12 months				0.33
≥ 1 Visit	170 (41.8)	51 (12.5)	119 (29.2)	
No Visit	237 (58.2)	55 (13.5)	182 (44.7)	
Adjunct teeth cleaning methods ^†^				0.24
Chewstick/*mswaki*	53 (13.0)	14 (3.4)	39 (9.6)	
Charcoal	37 (9.1)	8 (2.0)	29 (7.1)	
Wooden toothpick	23 (5.7)	3 (0.7)	20 (4.9)	

Note: * Significant to Chi-square test (< 0.05); ^**†**^ = Categories are not mutually exclusive, N = Frequency

**Fig. 1:**
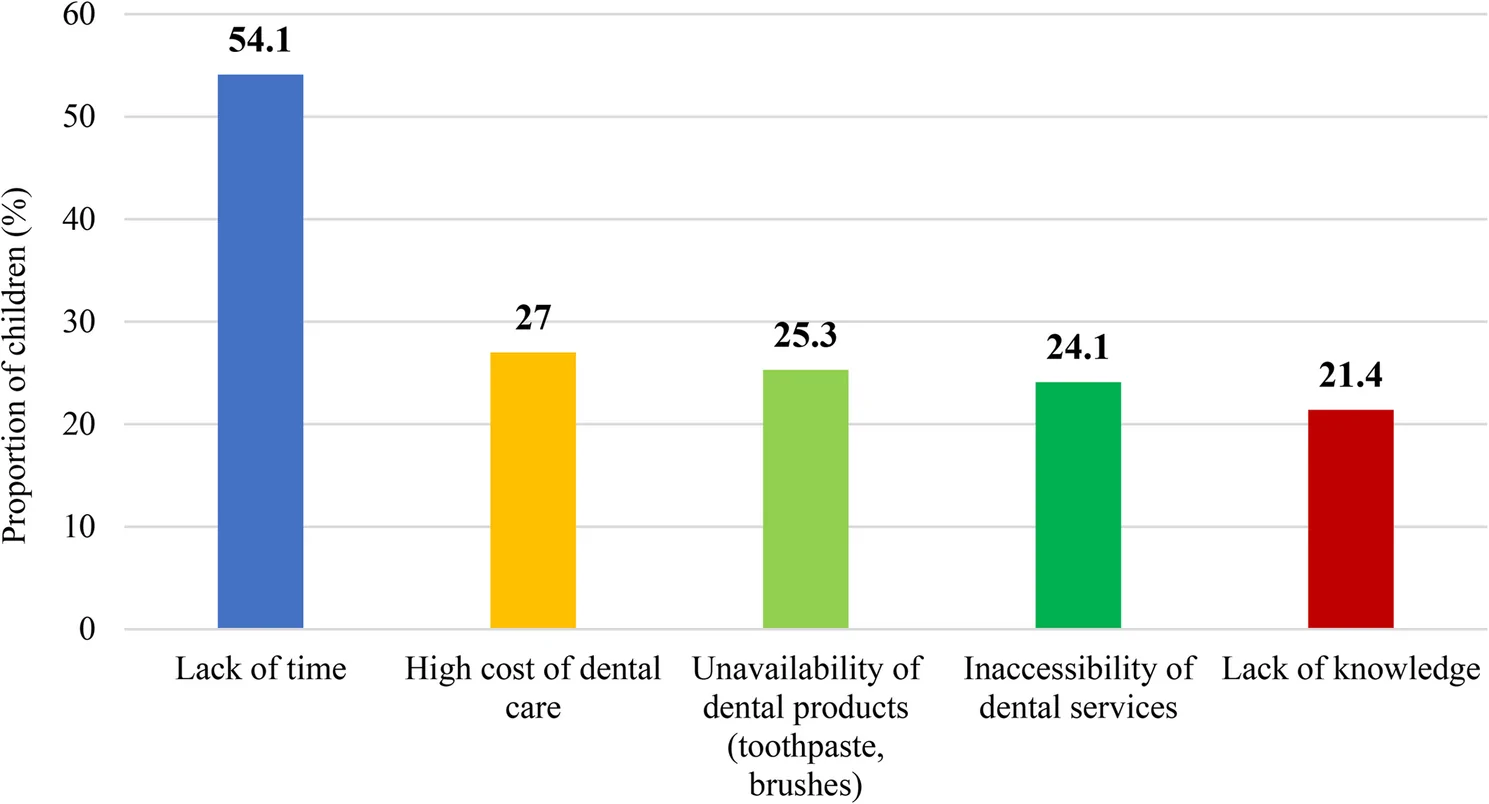
Common barriers to maintaining good oral hygiene among primary school children

Figure 1 shows common barriers to maintaining good oral hygiene reported by primary school children. More than half (54.1%) cited lack of time (attributed to rushing to school to avoid lateness) as the main barrier. Other key barriers reported included the high cost of dental care (27.0%), unavailability of dental products such as toothpaste and toothbrushes (25.3%), inaccessibility of dental services (24.1%), and inadequate knowledge on hygienic oral practices (21.4%).

Multivariate logistic regression models revealed daily intake of biscuits, cakes, sweet pies, and buns (AOR = 2.31; 95% CI: 1.36–3.74; *p* = 0.004); daily intake of soft drinks such as lemonade and Coca-Cola (AOR = 2.76; 95% CI: 1.39–4.53; *p* = 0.003); daily consumption of sweets and candy (AOR = 2.42; 95% CI: 1.35–3.91; *p* = 0.006); brushing less than twice daily (AOR = 2.92; 95% CI: 1.18–3.87; *p* = 0.023); and not using fluoride toothpaste (AOR = 1.83; 95% CI: 1.12–2.61; *p* = 0.043) were independently associated with higher odds of dental caries among primary school children. After multivariate adjustments, daily intake of milk or tea with sugar and brushing only once a day were not significantly associated with dental caries (Table 4).

**Table 4:** Dietary and Oral Hygiene Practices Associated with dental caries among primary school children

Variable	COR	95% CI	*p*-value	AOR	95% CI	*p*-value
Biscuits, cakes, cream cakes, sweet pies, buns, etc.						
≤ 1 times/week	Ref	–	–	Ref	–	–
2–6 times/week	1.42	0.93–2.27	0.18	1.35	0.83–2.20	0.26
Daily	2.67	1.51–4.89	< 0.001	2.31	1.36–3.74	0.004*
Lemonade, Coca Cola or other soft drinks						
≤ 1 times/week	Ref	–	–	Ref	–	–
2–6 times/week	1.57	0.79–2.84	0.083	1.48	0.88–2.49	0.23
Daily	3.18	1.69–5.17	< 0.001*	2.76	1.39–4.53	0.003*
Sweets/candy						
≤ 1 times/week	Ref	–	–	Ref	–	–
2–6 times/week	1.41	0.87–2.43	0.26	1.25	0.76–2.06	0.38
Daily	2.54	1.67–4.19	< 0.001*	2.42	1.35–3.91	0.006*
Milk with sugar						
≤ 1 times/week	Ref	–	–	Ref	–	–
2–6 times/week	1.31	0.58–2.17	0.071	1.20	0.64–1.75	0.19
Daily	1.95	1.23–4.09	0.038*	1.67	0.81–3.57	0.073
Tea with sugar						
≤ 1 times/week	Ref	–	–	Ref	–	–
2–6 times/week	1.25	0.71–2.08	0.69	1.07	0.58–1.69	0.74
Daily	1.67	0.85–2.53	0.13	1.38	0.64–2.31	0.33
Teeth cleaning frequency						
≥ 2 times/day	Ref	–	–	Ref	–	–
< 2 times/day	3.18	1.47–4.62	0.002*	2.92	1.18–3.87	0.023*
Fluoride Toothpaste Use						
Yes	Ref	–	–	Ref	–	–
No	1.98	1.13–2.79	0.026*	1.83	1.12–2.61	0.043*
Timing of Brushing						
Morning & Evening	Ref	–	–	Ref	–	–
Morning or Evening only	1.76	1.15–3.53	0.043*	1.39	0.65–2.25	0.31

*AOR* Adjusted Odds Ratio (multivariate logistic regression), *COR* Crude Odds Ratio (bivariate logistic regression), *Ref* Reference category

* = statistically significant *p* < 0.05

## Discussion

The findings of this study demonstrate that poor dietary patterns and oral hygiene in school children in informal settlements of Uasin Gishu County remain high and confirm the worrying trend of dental caries prevalence in school children in the region over the past few years [14]. To our knowledge, this is the first study on oral health and the combined influence of dietary patterns and oral hygiene practices on dental caries among schoolchildren residing in urban informal settlements. The current study observed a high frequency of sugar-containing food and beverage consumption among primary school children in informal settlements, which remains a significant public health concern in slum settings in Kenya. This finding is consistent with previous studies that reported persistent high daily intake of sugar-sweetened beverages and snacks among school-age youth [12, 13]. Intake of sugary foods and drinks among school-aged youth often exceeds WHO recommendations for free sugar consumption of less than 10% of total energy. A study in Nigerian informal settings reported that frequent intake of sweets, soft drinks, and sweetened beverages is a pervasive behaviour among school-aged children, particularly attributed to limited parental supervision, suboptimal nutrition and oral health literacy, and constrained availability and accessibility of healthy food [11]. Children living in low-resourced, densely populated settings are disproportionately exposed to and rely on cheap, readily available sugary snacks and beverages at an early age, which are often more affordable and accessible than healthy alternatives. This unhealthy dietary behaviour predisposes children to early-onset dental caries and long-term impact on QoL.

Consistent with previous studies, the current study observed a higher dental caries trend among children who reported daily consumption of sugary food and beverages [6, 10]. These findings demonstrated the sustained effect of high intake of sugary foods and beverages rather than sporadic exposure. According to Seemi et al. [20] dental caries trends among school-aged youths in informal settlements are amplified by aggressive marketing, low-cost sugary beverages, limited preventive dental services, higher poverty rates, and minimal regulatory control. This could explain why vast majority of dental caries remain untreated in informal settings. The observed trend demonstrates an existing inequitable distribution of oral health risk, where habitual sugar intake in deprived settings contributes to an increased burden of preventable diseases such as dental caries. Therefore, there is a need to implement integrated school- and community-based sugar reduction strategies focused on oral health education, regulation of sugary food availability, and strengthening of preventive oral health services within informal settlement settings.

The study observed suboptimal oral hygiene practices among school-aged children in informal settlement settings in Uasin Gishu County. Most primary school children reported inadequate toothbrushing, not using toothpaste, and a lack of fluoride toothpaste. These demonstrate structural and behavioural determinants of oral health hygiene among school children rather than isolated individual choices. Fluoride toothpaste remains the single most effective strategy to prevent caries incidence through remineralization of enamel and inhibition of bacterial metabolism [18]. Suboptimal utilization of fluoride toothpaste signals a significant primary preventive gap in efforts to address dental problems. Brushing twice daily with fluoridated toothpaste is the internationally recommended minimum standard for effective prevention of dental caries and other dental problems, including control of plague [18]. These findings are consistent with previous studies that reported suboptimal oral health practices in low-resource settings [12, 21]. Routine oral hygiene practices might be influenced by financial constraints, inaccessibility of primary oral health commodities, limited oral health literacy, and often minimal caregiver supervision. In most instances, affordability and availability often shape oral health behaviours in informal settlement settings rather than knowledge alone.

Additionally, persistent elevated dental caries prevalence among school children with suboptimal oral hygiene practices observed in this study is consistent with prior evidence [22, 23]. This could be attributed to exposure to cariogenic conditions due to prolonged plaque biofilm maturation and acidogenic activity. In Kenya, childhood oral health is primarily guided by the National Oral Health Policy [24], which emphasizes prevention through health education, fluoride use, and integration of oral health into primary healthcare and school health programs. However, implementation gaps remain, particularly in low-resource urban informal settlements, where access to preventive services and sustained oral health promotion is limited. Limited time, particularly in the morning on school days, was identified as a major barrier to consistent oral hygiene practices. Similar findings were reported by previous studies [25, 26]. This explains the need for good time management, such as establishing fixed morning hygiene routines at home, and integrating oral hygiene reminders within daily schedules, to ensure that children maintain consistent and effective tooth cleaning practices despite time pressures.

Daily consumption of sugary snacks and soft drinks independently doubled to nearly triple the odds of dental caries. These findings align with previous studies that reported higher odds of dental caries among school children with high intake of sugary foods [8, 10, 22]. A study in Nigeria reported that frequent sugar intake is a significant, strong predictor of dental caries among children [11]. Similarly, the current study observed that inadequate oral hygiene, particularly infrequent brushing and absence of fluoride toothpaste use, substantially increased children’s caries risk. These findings are consistent with previous studies conducted in Jordan [8], Johannesburg [10] and China [27]. Similarly, a study reported that brushing twice daily with fluorinated toothpaste significantly reduces odds of dental caries compared to infrequent brushing [23]. Our study demonstrates a critical need for tailored dietary risk reduction approaches integrated into school health programs. Despite this evidence, affordability and accessibility barriers to fluoride toothpaste, limited oral health awareness, and weak enforcement of school-based preventive programs constrain effective uptake in informal settlements. Consequently, there is a critical need for strengthened implementation of evidence-based interventions, including structured school oral health programs, integration of supervised toothbrushing initiatives, sustained dietary counseling targeting sugar reduction, and policy-driven measures to improve equitable access to affordable fluoride toothpaste. These interventions require coordinated action between health and education sectors to translate national policy into practical, context-specific strategies capable of improving oral health outcomes and quality of life among vulnerable school-aged children.

### Strengths and limitations of the study

The study had several strengths. This study fills existing data and knowledge gaps on dietary patterns and oral health practices among primary school children in informal settlement settings in Eldoret City, Kenya. To our knowledge, this is the first study to be done in informal settlement areas in the Eldoret city, hence critical to address behavioural gaps. The study adopted validated and customized questionnaires and other study tools such as WHO dental assessment guidelines [17]. The study was conducted across randomly selected primary schools, promoted a wide representation. Integration of clinical dental caries assessment alongside with self-reported practices provided both subjective and objective assessment, enhanced internal validity. Additionally, the study’s findings provide context-specific programmatic recommendations suitable for informal settlement settings in urbans, which are underrepresented in global oral health research.

The study also had its limitations. First, the study adopted cross-sectional design, thus unable to establish causal-effect relationship between dietary patterns, oral hygiene practices, and caries. Secondly, reliance of self-reported dietary and oral hygiene behaviours could have been influenced by recall and social desirability bias, potentially overestimating or underestimating behaviours. Thirdly, clinical assessments were limited to observable caries without radiographic confirmation, possibly under-detecting interproximal lesions. Lastly, the study was conducted in informal settlement settings and might not be generalizable to schoolchildren in rural or urban areas.

## Conclusion

This study reveals a high burden of dental caries among primary school children in informal settlements, driven by daily consumption of sugary foods and beverages and inadequate oral hygiene practices. Frequent intake of sugary snacks and soft drinks independently increased caries risk, while brushing less than twice daily and non-use of fluoride toothpaste further amplified vulnerability. The findings of this study demonstrate sustained exposure to preventable and modifiable behavioural risks within structurally disadvantaged environments. Targeted school-based sugar reduction policies, supervised toothbrushing programs, and equitable access to affordable fluoridated toothpaste are urgently required to mitigate preventable disease and reduce oral health inequities in marginalized urban populations.

## Data Availability

The datasets generated and/or analyzed during the current study are available from the corresponding author on reasonable request.

## Abbreviations

AOR: Adjusted odds ratio
CDQ: Children’s dietary questionnaire
COR: Crude odds ratio
OHI-S: Simplified oral hygiene index
SPSS: Statistical Package for the Social Sciences
QoL: Quality of life
